# The Role of Feature Tracking in the Furrow Illusion

**DOI:** 10.3389/fnhum.2016.00081

**Published:** 2016-03-07

**Authors:** Rémy Allard, Jocelyn Faubert

**Affiliations:** ^1^Sorbonne Universités, Pierre and Marie Curie University Paris 06, Institut National de la Santé et de la Recherche Médicale, Centre National de la Recherche Scientifique, Institut de la VisionParis, France; ^2^Visual Psychophysics and Perception Laboratory, Université de MontréalMontréal, QC, Canada

**Keywords:** furrow illusion, motion, periphery, feature tracking, crowding, attentional resolution

## Abstract

In the furrow illusion (Anstis, 2012), the perceived path of a moving target follows the veridical path orientation when viewed foveally, but follows the orientation of the texture when viewed peripherally. These radically different motion percepts depending on whether the stimulus is viewed foveally or peripherally has led Anstis to conclude that the furrow illusion reveals “profound differences in the way that the periphery and fovea process visual motion.” In the current study, we rather argue that the different percepts can be explained by reduced position acuity with eccentricity and therefore do not imply different ways of processing motion *per se*. If feature tracking, which is position-based, is involved in the perception of the veridical motion direction, then impairing the feature tracking motion system should strengthen the illusion. To reduce contribution of the feature tracking motion system, we used a crowding paradigm consisting in presenting many nearby targets. We found that under crowding conditions, the furrow illusion was stronger. We conclude that feature tracking was involved in the perception of the veridical motion direction, which is compatible with the hypothesis that the different motion percepts at fixation and in the periphery are due to a reduced position acuity with eccentricity affecting feature tracking, not to different ways of processing motion *per se*.

## Introduction

In the furrow illusion (Anstis, 2012), the perceived path of a moving spot is radically different depending on whether the spot is viewed foveally or peripherally. This difference could be due to different ways of processing motion with eccentricities or to pre-motion processing factors that vary with eccentricity. An important factor that changes with eccentricity is visual acuity (Anstis, 1974, 1998), but artificially reducing visual acuity at the fovea with blur did not result in the peripherally perceived path (Anstis, 2012). Anstis concluded that the furrow illusion reveals “profound differences in the way that the periphery and fovea process visual motion” (p.10). However, there are other differences in the peripheral processing of visual information. Notably, crowding is a well-known phenomenon that impairs object recognition in the periphery (Pelli et al., 2004; Levi, 2008). Although the specific underlying factors responsible for crowding remains debated, it is well-established that crowding is not due to visual acuity and is not specific to motion processing *per se*. The present article investigates if the processing factor responsible for crowding could explain the difference in the perceived motion direction with eccentricity in which case the radically different motion percepts when foveally and peripherally viewing the furrow illusion would not be caused by different ways of processing visual motion *per se*.

**Figure 1:**
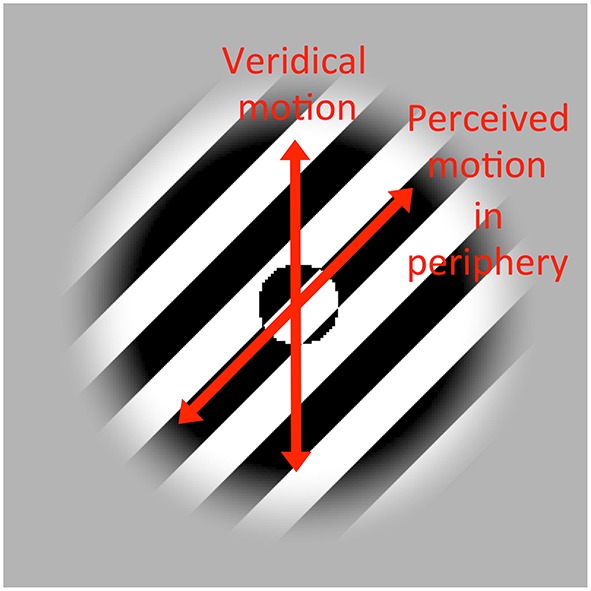
**Furrow illusion**. When a spot (here a negative lens inverting contrast polarity) drifts over an oriented texture, the perceived motion path at fixation usually matches the veridical motion path of the spot (here vertical), but when the same stimulus is viewed peripherially, the spot is perceived as moving along the texture orientation. To experience the illusion, see Movie 1 in Supplementary Material.

In the furrow illusion, one of the conditions under which the perceived path differs the most between central and peripheral viewing is when the background texture is a static black and white square-wave and a spot operating as a negative lens inverting contrast polarity drifts along a path that differs by 45° from the texture orientation (Figure 1). At fixation, the spot is perceived as moving along its veridical path, whereas in the periphery, it is illusory perceived as moving along an path parallel to the texture orientation, i.e., a perceptual error of 45°, which “may be the strongest directional illusion known” (p.5, Anstis, 2012).

In the different versions of the furrow illusion (Anstis, 2012) and many other illusions (e.g., Mather et al., 1985; Tse and Hsieh, 2006; Shapiro et al., 2010, 2011), the perceived motion path of the spot differs depending on whether it is viewed foveally or peripherally. These illusions have in common the fact that the direction of the mean local motion signal does not match the direction of the position displacement of the spot. At fixation, the shape is generally perceived as moving along its veridical global shape-defined path, whereas in the periphery it is perceived as moving along a path biased toward the direction of the mean local motion signal. Thus, the illusory perceived path in the periphery is undoubtedly driven by local motion signals. In the furrow illusion, local motion signals follow, on average, the orientation of the texture and not the orientation of veridical shape-defined path (Anstis, 2012).

If perceived position displacements of the spot in the furrow illusion are computed from the local motion signals, then the illusory position displacements in the periphery can easily be explained as being inferred from a simple averaging of local motion signals and, paradoxically, it is the veridical perceived position displacements at fixation that are less trivial to explain and more controversial. At fixation, the perceived path cannot be inferred from a simple averaging of local motion signals; there must be some other process involved that enables the visual system to properly extract the veridical position displacements. One possibility is a complex computational combination of local motion signals. Anstis suggested that the fovea could discriminate whether local motion signals belongs to the moving target or to sliding intersections between the target edges and the background due to differential processing of local velocity differences (Braddick, 1993) and a balanced combination of integration and computationally expensive differentiation would enable the recovery of veridical position displacements.

An unlikely implication of this interpretation is that the foveally perceived *position* would be inferred from a temporal integration of complex computations of local motion signals rather than by directly localizing the global shape of the spot based on its spatial information. If the perceived path (i.e., perceived position of the shape over time) is computed solely from local motion signals, this suggests that the perceived position at a given time is computed from the previously perceived position shifted by a displacement computed from local motion signals. Given that the direction of the mean local motion signal deviates by 45° from the direction of veridical position displacements, retrieving veridical position displacements at fixation would require complex and expensive computation as suggested by Anstis. As a result, the perceived position at a given time would be the result of a temporal integration of position displacements each being the result of a complex computation of local motion signals. But localizing the shape at fixation based on the temporal integration of complex derivatives (i.e., perceived position displacements) seems unlikely since the shape can be localized directly and precisely based on its spatial information (its shape is clearly visible statically as in Figure 1). We therefore suggest that that the veridical perceived position at fixation is inferred from localizing the spot based on spatial information rather than by temporally integrating position displacements based on a complex computation of local motion signals.

In the periphery, however, position acuity is low (Klein and Levi, 1987; Levi et al., 1987; Morrone et al., 1989; Levi and Waugh, 1994). Given imprecise position estimates based on spatial information, the perceived position at a given time would be computed from the previously perceived position shifted by a displacement inferred from a simple averaging of local motion signals. Given that the mean direction of local motion signals matches the texture orientation, the spot would be perceived along this illusory path.

More generally, we suggest that the perceived position of the spot depends on two factors: the likelihood of the position estimate based on spatial information and the averaging of local motion signals suggesting the direction of the position displacement. When the position estimate is precise such as at fixation, the likelihood of the position estimate based on spatial information would be finely tuned around the veridical position and local motion signals would have negligible impact on the perceived position (and thereby on the perceived path). The influence of local motion signals on the perceived position would be greater when the likelihood of the position estimate is broader based on spatial information (i.e., lower certainty of position). As a result, the perceived path would increasingly be biased along the texture orientation with eccentricity where position estimates are less precise.

This interpretation does not necessitate computationally expensive processing of local motion signals to explain the perceived veridical motion path at fixation, because it could simply consists in attentively tracking the change in position of the global shape (i.e., a spot). It is well-known that we have at least two motion systems: a low-level, energy-based motion system (Adelson and Bergen, 1985; van Santen and Sperling, 1985; Watson and Ahumada, 1985) that is sensitive to luminance-defined motion^1^ and is fast (temporal cutoff frequency around 12 Hz, Lu and Sperling, 1995) and a high-level, feature tracking motion system (Cavanagh, 1992) that can track many features and is slow (cutoff frequency around 3 Hz, Lu and Sperling, 1995). The feature tracking motion system consists in attentively tracking a change in position of a given feature. In the furrow illusion, the feature is a spot operating as a negative lens, which does not need any motion processing to be precisely localized at fixation, as it is clearly visible statically (Figure 1). As a result, tracking the global shape (here, a spot) at fixation would not depend on local motion signals and would therefore extract the veridical motion direction as observed foveally in the furrow illusion (Anstis, 2012). Thus, we suggest that the simplest interpretation of the furrow illusion is that the perceived motion path of the spot was determined by the feature tracking motion system foveally (by tracking the shift in position of the shape) and by the energy-based motion system peripherally (by averaging local motion signals and biasing the perceived position).

The main objective of the present study was to investigate whether the perceived veridical path at fixation in the furrow illusion is due to the energy-based motion system through computationally expensive processing of local motion signals (as suggested by Anstis, 2012) or to the feature tracking motion system attentively tracking the position of the shape. According to the energy-based hypothesis, the local motion signals would be used differently at the fovea and in the periphery: integration would dominate in the periphery and a combination of integration and differentiation would operate foveally, so, there would be fundamentally different ways of processing local motion signals in the periphery and foveally. According to the feature tracking hypothesis, the shift in ways of processing motion between fixation and periphery would not be attributed to different ways of processing motion processing *per se*, but to a pre-motion processing factor affecting the likelihood of position estimates. The current study tested this hypothesis using a crowding paradigm to impair feature tracking (Allard and Faubert, 2013a). If the veridical motion depends on the feature tracking motion system and illusionary motion percept is due to averaging of local motion signals by the energy-based motion system, then impairing the feature tracking motion system should strengthen the illusion.

## Methods

### Observers

Ten naïve observers (5 females and 5 males) aged between 21 and 38 participated in this study. They had normal or corrected-to-normal vision. Ethical approval was obtained from the University's ethics board and written informed consent was obtained from the participants.

### Apparatus

The stimuli were presented on a 22.5-inch LCD monitor designed for psychophysics (VIEWPixx) with a refresh rate of 120 Hz. At the viewing distance of 43 cm, the width and height of the screen were 69 and 39° of visual angle (dva). The monitor was the only source of light in the room. The output intensity of each color gun was linearized psychophysically using a homemade program.

### Stimuli and procedure

**Figure 2:**
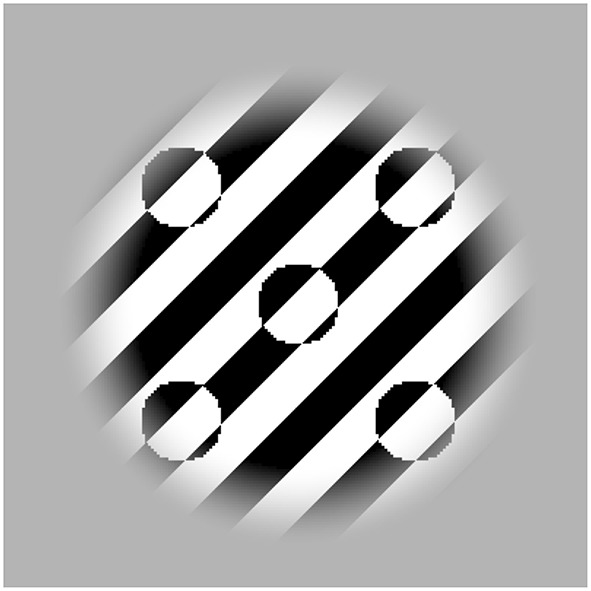
**Stimlus composed of five spots moving in a phase-lock configuration**. When viewed peripherally, it should be harder to attentionally select one, which should compromise (or reduce) the ability to track them. To experience the lower ability to attentively select them, try counting them when viewed peripherally. See Movie 2 in Supplementary Material.

Instead of presenting a single spot that can easily be selected and localized by attention even when attention resolution is low (Figure 1), many nearby spots were presented and moved together in a phase-lock configuration (Figure 2). Feature tracking requires to attentively select a feature (here, a spot) before tracking its change in position. Given that adding nearby flankers impairs the ability of attention to select a target in the periphery due to low attention resolution (He et al., 1996; Intriligator and Cavanagh, 2001), feature tracking should become difficult when attentional resolution is too low to select and localize individual features thereby impairing its contribution to motion (Allard and Faubert, 2013a). Thus, by presenting many nearby objects, the motion percept should rely more on energy-based motion processing, which should strengthen the illusion. Conversely, if the furrow illusion is due to a change in the way local motion signals are combined, we would not expect that adding targets should change the relative weight of these processes so that it should have little impact on the furrow illusion.

A crucial concept for the purpose of the present study is that feature tracking would be impaired when adding nearby spots. Nonetheless, some readers may still be skeptical. To directly demonstrate the impact of adding nearby spots on feature tracking, consider Movies 3, 4 in Supplementary Material. These movies are identical to Movies 1, 2 in Supplementary Material, respectively, with the exception that any contribution of the energy-based motion system was neutralized by removing two frames out of three introducing 67 ms gaps (Smith, 1994). As a result, with Movie 3 in Supplementary Material, motion along the veridical path orientation is perceived even at far eccentricities, which shows that the spot can be attentively tracked. With Movie 4 in Supplementary Material, however, the perception of motion is veridical at fixation but much less obvious in the periphery. This demonstrates that adding the four flankers impaired the feature tracking motion system peripherally, but not foveally, which is consistent with our previous findings (Allard and Faubert, 2013a,b). If it is the feature tracking motion system that is responsible for the veridical perceived motion direction in the furrow illusion, then impairing feature tracking should strengthen the illusion.

The background texture was a black and white square wave with a spatial frequency of 1 cycle per dva and its orientation was randomized at each trial. The spot operated as a negative lens inverting the contrast polarity of the texture. Its shape was circular with a diameter of 1 dva and it oscillated back and forth along an axis that differed from the background by 45° (either clockwise or counter-clockwise, Figure 1). The speed of the spot was constant at 6 dva/s and the amplitude of the motion (peak-to-peak) was fixed to 2 dva resulting into a moving stimulus at 1.5 Hz. The stimulus location (mean spot position) was either −18, −12, −6, 0, 6, 12, or 18° of eccentricities from fixation along the horizontal axis. A spatial window modulating the texture had a diameter of 4 dva plus a halfcosine ramp of 1 dva and was always centered on the mean target location. In the crowding condition (Figure 2), four spots were added 2 dva from the center spot (center-to-center distance) as shown in Figure 2 and all the spots moved in a phase-lock configuration (Movie 2 in Supplementary Material).

Observers were asked to fixate at a bar presented at the center of the screen and adjust its orientation by controlling a computer mouse so that it matched the orientation of the perceived path. When more than one spot were presented, observers were asked to report the orientation of the perceived path of the central spot. The observers were instructed that if they ever shifted their gaze position during a trial, they should fixate back at the central bar and report the orientation of the perceived path when fixating the bar. When the observer was satisfied with the adjustment, he/she pressed a mouse button to record his/her answer. The bar was 50 pixels (i.e., 1.8 dva) long and 5 pixels wide (i.e., 0.18 dva). It was white with a 1-pixel wide black on its circumference and a 3-pixel wide fixation point was added to its center. When the stimulus was shown at fixation, the bar appeared on top of the stimulus. Note that this made the task trivial, but the important conditions are the ones in the periphery where the illusion is seen and it is obvious that we perceived the veridical motion when gazing at the stimulus (Anstis, 2012, this can also be experience directly by gazing at Movies 1, 2 in Supplementary Material). The next trial was automatically initiated 500 ms after the answer was reported. The stimulus remained visible until the observer responded.

There were 28 different stimulus conditions that were tested twice in a pseudo-random order: 7 eccentricities, 2 directions (45° clockwise or counter-clockwise from the background orientation) and 2 crowding conditions (1 or 5 spots).

### Data analysis

For each trial, the reported orientation was normalized relative to the drifting orientation so that 0° represented the veridical drifting orientation of the spot and 45° represented the texture orientation. Data were averaged across absolute eccentricities (i.e., left and right) and drifting orientation (+/− 45°). As a result, the estimated orientation of the perceived path at fixation for a subject corresponded to the average of 4 trials (2 drifting directions performed twice) and the other data points were averaged across 8 trials due to the merged negative and positive eccentricities. These averaged data were analyzed with a 4 × 2 ANOVA (eccentricity × crowding condition).

## Results

**Figure 3:**
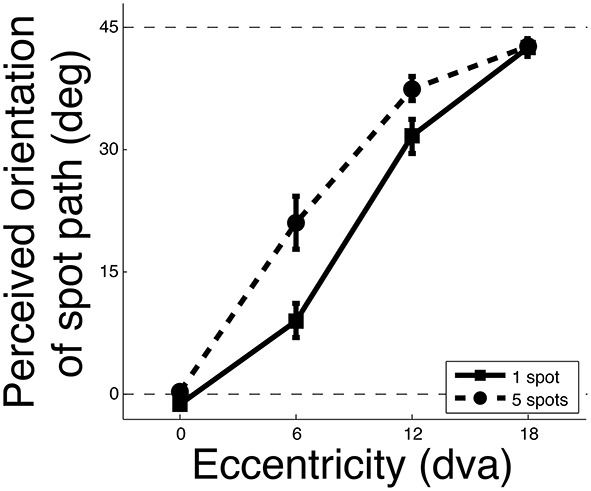
**Strength of the illusion as a function of eccentricity**. The orientation of 0° corresponded to the veridical motion orientation as viewed foveally (eccentricity = 0). The orientation of 45° corresponded to the background orientation. The perceived motion orientation was more influenced by the background orientation in the 5-spot configuration (dashed line) compared to the 1 spot configuration (solid line), i.e., the illusion was stronger. Error bars represent standard errors.

A significant interaction between eccentricity and crowding condition was observed [*F*_(3, 27)_ = 10.6, *p* < 0.001] showing that different crowding effects (1 vs. 5 spots) were observed at different eccentricities. Specifically, when presenting 5 moving spots instead of 1, the furrow illusion was strengthened, that is, the stimulus had to be presented at smaller eccentricities to perceive motion following the texture orientation (Figure 3). This was statistically confirmed using paired *T*-tests as the perceived orientation at 6 and 12° of eccentricity were significantly higher when presenting 5 spots instead of 1 [*t*_(9)_ = 4.9 and 3.6 and, *p* < 0.001 and 0.01, respectively]. Thus, presenting many nearby spots instead of one strengthened the illusion. Given that the 5-spot configuration impaired feature tracking, the strengthened of the furrow illusion suggests that it is the feature tracking that was responsible for the perception of the veridical motion direction.

Given that this experiment evaluated the strength of the illusion as a function of eccentricity, a crucial factor to consider is the eccentricity span of the stimulus, which was larger with 5 spots compared to 1. However, even if observers did not report the path orientation of the central spot (as instructed), but rather relied on anyone of the 5 spots (or relied on different spots at different trials), this could not explain the stronger effect with 5 spots. The illusory effect observed with 5 spots at 6 and 12° of eccentricity is equivalent to the illusory effect observed with 1 spot at about 9.2 and 15.3° of eccentricity, respectively, (assuming linear interpolation between the illusory strength observed with 1 spot). However, the flankers were only, at the most, 2° further in eccentricity than the central spot. So even if observers (surprisingly) based their judgment only on the furthest flanker (the one that would generate the strongest illusion), the illusion would still be too weak to explain the illusion observed with 5 spots. We therefore conclude that the stronger illusory effect with 5 spots cannot be explained by the fact that observers reported the motion direction of a spot other than the central one; it must be due to processing interference of nearby spots, i.e., crowding.

**Figure 4:**
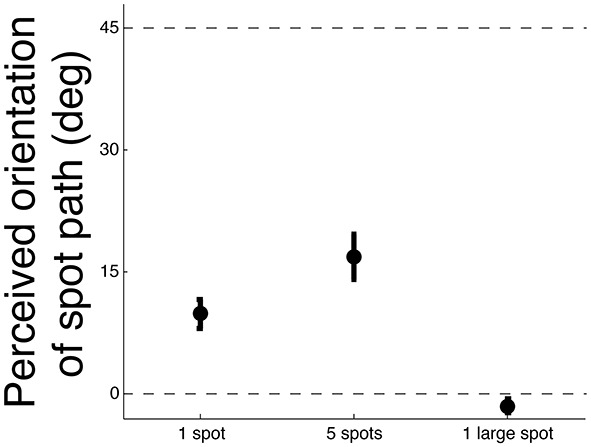
**Strength of the illusion for the control experiment at 6° of eccentricity for 3 conditons: 1 spot (diameter of 1 dva), 5 spots (each with a diameter of 1 dva), and 1 large spot (diameter of 2.2 dva)**. As in Figure 3, the orientation of 0° corresponded to the veridical motion orientation as viewed foveally (eccentricity = 0). The orientation of 45° corresponded to the background orientation. Error bars represent standard errors.

It is also possible that the illusion was stronger when the total area of the stimulus was larger (i.e., with 5 spots instead of 1) rather than due to a lower contribution of the feature tracking motion system. To control for this confound, an experiment was performed at the eccentricity showing the greatest effect (i.e., 6°). The strength for the illusion was measured for 1 spot having an area greater by a factor of 5 (i.e., diameter of 2.2 dva instead of 1). All other parameters remained the same. To enable direct comparison, the conditions with 1 and 5 spots with diameters of 1 dva were also retested. The results averaged over 7 observers are plotted in Figure 4. Replicating the previous findings at 6° of eccentricity (Figure 3), the strength of the illusion was greater with 5 spots instead of 1 as confirmed by a paired *T*-test [*t*_(6)_ = 4.2, *p* < 0.01]. Controlling for the spot area by presenting only one spot covering the same area as the five spots did not increase the strength of the illusion; conversely, it abolished it [*t*_(6)_ = 6.7, *p* < 0.01]. Thus, the strengthening of the illusion with 5 spots instead of 1 cannot be explained by the larger total area of motion. The weakening of the illusion when increasing the spot size can be explained by the fact that large objects were easier to track in the periphery as the edges of the spot are further apart from one another.

## Discussion

Presenting 5 nearby spots moving in block instead of 1 in the near periphery made the orientation of the perceived motion path closer to the texture orientation. Given that presenting nearby elements in the periphery impairs feature tracking (Allard and Faubert, 2013a), this strengthening of the furrow illusion suggests that the perception of the veridical motion direction at fixation was due to the feature tracking motion system. Given that the shape of the spot can be perceived without any motion information (e.g., the circular shape of the spot is clearly visible statically as seen in Figure 1), this motion system does not need to rely on the local motion signals. Directly tracking the change in position of the spot is sufficient to extract its veridical motion path. A computationally expensive combination of local motion signals is not required.

In the present study feature tracking was impaired using a crowding paradigm analogous to the one we have used for gratings (Allard and Faubert, 2013a). The rationale of this paradigm was that feature tracking is attention-based and therefore should be impaired when preventing attention from selecting and localizing individual features such as when presenting many nearby elements in the periphery where attention resolution is low (He et al., 1996; Intriligator and Cavanagh, 2001). In our previous study (Allard and Faubert, 2013a), feature tracking was impaired by presenting many nearby elements (bars) all moving as a block in the same direction. When the elements were sparse enough for attention to select some (i.e., spatial frequencies below attentional resolution), the bars could be attentively tracked. But when the elements were too close one to another to be selected by attention (i.e., spatial frequencies above attentional resolution), tracking them became impossible, as they did not contribute to motion perception. Tracking them individually or as a group would have resulted in a contribution to motion perception so having many clearly visible elements moving as a block prevented attention from tracking them individually and as a group. The present study found that impairing the feature tracking motion system by presenting many nearby elements strengthen the illusion, which suggests that the illusory motion percept in the periphery was due to a failure of the feature tracking motion system and the perceived veridical motion near fixation was therefore due to the feature tracking motion system.

Note that this interpretation of the feature tracking impairment when presenting many nearby elements in the periphery assumes that crowding is due to a limited attentional resolution as suggested by many studies (e.g., He et al., 1996; Intriligator and Cavanagh, 2001; Strasburger, 2005; Põder, 2006; Fang and He, 2008; Petrov and Meleshkevich, 2011; Chen et al., 2014), but which remains controversial (e.g., Pelli et al., 2004; Freeman and Pelli, 2007; Levi, 2008; Dakin et al., 2009; Greenwood et al., 2009). For instance, Dakin et al. (2009) found a double dissociation between attention and crowding suggesting that they involve distinct neural mechanisms, but their interpretation has been questioned (Allard and Cavanagh, 2012). Nonetheless, even if crowding was not due to low attentional resolution, crowding would still be expected to impair the position-based motion system (i.e., feature tracking) because it induces position uncertainty (Freeman and Pelli, 2007). Another concern could be that the strengthening of the illusion is not due to crowding, but to the fact that observers tracked the global configuration as a unitary percept. However, the position uncertainty of a group is lower than the one of an individual element in the periphery (Levi et al., 1987), so this interpretation also predicts that feature tracking would be impaired when presenting many nearby elements in the periphery. In any case (low attentional resolution, crowding or tracking global configuration), having many elements moving as a block is expected to impair feature tracking in the periphery as previously observed (Allard and Faubert, 2013a) and this was confirmed in the Methods section above, which suggests that the strengthening of the furrow illusion when adding many nearby dots was due to an impairment of the feature tracking motion system.

Although determining the exact cause of the feature tracking impairment has little importance for the purpose of the present study, we can speculate. It is unlikely that observers in the current study tracked the global 5-spot configuration as a unitary percept because the 5 spots were not always perceived as moving in the same direction. Some observers spontaneously mentioned that during some trials the 5 spots were perceived as moving in different directions. This can be experience by gazing at the bottom left corner of Movie 2 in Supplementary Material: the spot closest to this corner is perceived as moving in a different direction (closer to the veridical path) compared to the others (as confirmed by 5 naïve observers). Furthermore, when asking these naïve observers whether the two top spots were perceived as converging or diverging when gazing slightly to the left of the movie, they reported diverging when moving upwards and converging when moving downwards. They observed the opposite when fixating to the right, which is consistent with the fact that the illusion is stronger at larger eccentricities. This shows that the 5 spots were not always perceived as a unitary percept, which suggests that perceiving the 5 spots as a unitary percept is unlikely the cause of the impairment of the feature tracking. This leaves two hypotheses of the feature tracking impairment in the 5-spot configuration: low attentional resolution or crowding. If crowding is due to a limited attentional resolution, these two hypotheses can be seen as equivalent, but some argue that crowding is not related to attention. The data presented here will certainly not resolve this ongoing debate. Nonetheless, given that feature tracking is directly related to attention, the limited resolution of attention in peripheral vision is the more direct and most parsimonious explanation. So we favor this hypothesis.

Interestingly, there is another illusion also created by Anstis (1970) in which the mean local motion signals does not follow the veridical motion direction: reverse-phi motion. In this illusion, reversing the contrast polarity can result in perceiving motion in the opposite direction. Thus, if the perceived motion direction were based on the integration of local motion signals, the perceived motion direction would be opposite to the veridical motion. Presenting only two successive frames in which an object moves from one location to another and reverses its contrast polarity between the two frames is sufficient to create this illusion, but the illusion can also be presented as a continuous sequence of frames in which the contrast of a moving object is reversed at every frame. This continuous reverse-phi illusion is optimal when presenting many nearby objects in the near periphery (see Movie 5 in Supplementary Material, which was adapted from an unpublished version from Patrick Cavanagh). These conditions are optimal to prevent attention from selecting and tracking an object thereby compromising the contribution of the feature tracking motion system. When gazing at a continually moving object in which its contrast is reversed at every frame, the illusion usually disappears (e.g., gazing at an element in Movie 5 in Supplementary Material). Thus, the continuous reverse-phi motion can be analogous to the furrow illusion: in the periphery, motion is seen in the mean direction of the local motion signals and at fixation, the motion is perceived in the veridical direction.

Reversing the contrast polarity between two frames has the consequence of also reversing the direction of the motion energy. As for the furrow illusion, the illusion is easily explained by averaging local motion signals by the energy-based motion system. Again, the explanation of the condition under which motion is perceived in the veridical direction is less trivial. Given that the local motion energy is reversed, it is improbable to suggest that some complex combination of local motion signals can recover the veridical motion direction. The simplest interpretation is that the perceived motion in the veridical direction is determined by the feature tracking motion system, which does not depend on local motion signals, but on a position shift of a feature (e.g., shape). This interpretation is supported by the fact that the strength of the illusion in the periphery is reduced (or abolished) by gazing at a moving object or by presenting fewer objects enabling attention to select and track them (e.g., Movie 6 in Supplementary Material). Indeed, reducing the number of elements in the periphery enables the feature tracking motion system to contribute to motion processing, which results in the perception of veridical motion even in the periphery. This interpretation is also supported by the fact that adapting to reverse-phi motion causes a motion aftereffect in the *same* direction as the veridical shape-defined motion (i.e., opposite to local motion signals) even when the perceived motion is in the direction of the veridical shape-defined motion such as at fixation (Anstis and Cavanagh, 1981). This motion aftereffect led Anstis and Cavanagh to conclude that the perceived motion in the veridical shape-defined direction is due to a motion system that is not based on local motion signals. Consistent with this interpretation, we suggest that reverse-phi motion occurs when the perceived motion direction is determined by the averaging of the local motion signals and does not occur when it is determined by attentively tracking the shape, which dominates at fixation. This interpretation based on two motion systems, which seems to be the most likely to explain the fact that reversing contrast polarity does not always reverse the perceived direction and the motion aftereffect, can also explain the perceived veridical motion direction in the furrow illusion, e.g., when gazing a spot drifting over an oriented background. If the veridical motion percept in the furrow illusion is also due to feature tracking, then, as for the reverse-phi motion, motion aftereffect should also be perceived in the direction opposite to the mean local motion signals (i.e., along the texture orientation), even if the perceived motion path rather follow the veridical global shape-defined motion such as when viewed near fixation. A simple demo (notice the motion direction of the aftereffect after fixating at the central gray dot of Movie 7 in Supplementary Material) confirms this prediction: the motion is clearly perceived vertically (i.e., down and up along the veridical motion direction), but the motion after effect is not opposite to the perceived motion, it is oblique as the spots are drifting away from fixation along the texture orientation, that is, the motion aftereffect is opposite to the mean local motion signals. As suggested by Anstis and Cavanagh (1981) for reverse-phi, this suggests that the motion system responsible for the perceived motion differs from the one responsible for the motion aftereffect and therefore supports the claim that the veridical motion percept in the furrow illusion is due to the feature tracking motion system.

Furthermore, there are other illusions in which the perceived motion direction varies with eccentricity. These illusions have in common the fact that the direction of the mean local motion signals and the global shape-defined motion differs, and the perceived motion direction is biased toward the mean local motion signals as eccentricity is increased. Mather et al. (1985) created a stimulus in which the shape of a large bar was displaced in one direction, but the light/dark edges were displaced in the opposite direction. Foveally, the stimulus was more likely perceived as moving in the direction of the shape-defined motion whereas in the periphery it was more likely perceived as moving in the opposite, energy-based direction. Others (Tse and Hsieh, 2006; Shapiro et al., 2010, 2011) create illusions in which a sine wave and its aperture were drifting in different directions. When gazing at the stimulus, the perceived motion direction corresponds to the motion defined by the global shape of the stimulus (i.e., aperture), but the perceived motion direction was biased toward the drifting sine wave direction as the stimulus was presented further away from fixation. Our interpretation of the furrow illusion is compatible with all these illusions: feature tracking dominates when attention resolution is good (i.e., at fixation), but the influence of mean local motion signals processed by the energy-based motion system increases with eccentricity as the ability to attentively select and localize a target is impaired by presenting many nearby elements. Thus, the different motion percepts at fixation and in the periphery can be due to lower attentional resolution with eccentricity, and therefore do not imply fundamentally different motion processes *per se*.

The current study suggests that under low position uncertainty (e.g., when gazing at a moving object) the perceived position would be determined by the spatial information (i.e., global shape of the target) rather than through complex computational combination of local motion signals processed by the energy-based motion system. Under high position uncertainty, however, such as in the periphery and under crowding conditions, the perceived position would be biased by the mean local motion signal processed by the energy-based motion system. As a result, the feature tracking motion system would be responsible for the veridical perceived motion path at fixation and the energy-based motion system averaging local motion signals and affecting perceived position would be responsible for the illusory perceived path in the periphery. Nonetheless, given that the ability to localize and track features in the periphery is impaired due to lower position acuity, which is not specific to motion processing *per se*, the drastically different motion percepts observed foveally and peripherally do not necessarily imply different ways of processing motion *per se*.

## Author contributions

All authors listed, have made substantial, direct and intellectual contribution to the work, and approved it for publication.

### Conflict of interest statement

The authors declare that the research was conducted in the absence of any commercial or financial relationships that could be construed as a potential conflict of interest.

## Footnotes

^1^Note that some studies suggested that we have two energy-based motion systems: a first-order system sensitive to luminance-defined motion and a second-order motion system sensitive to texture-defined motion (e.g., Lu and Sperling, 1995, 2001; Scott-Samuel and Georgeson, 1999). However, recent studies strongly suggest that there is no energy-based, second-order motion system (Allard and Faubert, 2013a,b) so we only consider the first-order motion system sensitive to luminance-defined motion.
